# Evaluation of focus and deep learning methods for automated image grading and factors influencing image quality in adaptive optics ophthalmoscopy

**DOI:** 10.1038/s41598-021-96068-2

**Published:** 2021-08-17

**Authors:** Danuta M. Sampson, David Alonso-Caneiro, Avenell L. Chew, Jonathan La, Danial Roshandel, Yufei Wang, Jane C. Khan, Enid Chelva, Paul G. Stevenson, Fred K. Chen

**Affiliations:** 1 Centre for Ophthalmology and Visual Science (Incorporating Lions Eye Institute), The University of Western Australia, Perth, WA 6009 Australia; 2 Surrey Biophotonics, Centre for Vision, Speech and Signal Processing and School of Biosciences and Medicine, The University of Surrey, Guildford, GU2 7XH UK; 3 Contact Lens and Visual Optics Laboratory, Centre for Vision and Eye Research, School of Optometry and Vision Science, Queensland University of Technology (QUT), Kelvin Grove, QLD 4059 Australia; 4 Department of Computer Sciences, University of Wisconsin-Madison, 1210 W Dayton St, Madison, WI 53706 USA; 5 Department of Ophthalmology, Royal Perth Hospital, Perth, WA 6000 Australia; 6 Department of Medical Technology and Physics, Sir Charles Gairdner Hospital, Nedlands, WA 6009 Australia; 7 Telethon Kids Institute, The University of Western Australia, Perth, WA 6009 Australia; 8 Perth Children’s Hospital, Nedlands, WA 6009 Australia

**Keywords:** Imaging and sensing, Translational research, Health care

## Abstract

Adaptive optics flood illumination ophthalmoscopy (AO-FIO) is an established imaging tool in the investigation of retinal diseases. However, the clinical interpretation of AO-FIO images can be challenging due to varied image quality. Therefore, image quality assessment is essential before interpretation. An image assessment tool will also assist further work on improving the image quality, either during acquisition or post processing. In this paper, we describe, validate and compare two automated image quality assessment methods; the energy of Laplacian focus operator (LAPE; not commonly used but easily implemented) and convolutional neural network (CNN; effective but more complex approach). We also evaluate the effects of subject age, axial length, refractive error, fixation stability, disease status and retinal location on AO-FIO image quality. Based on analysis of 10,250 images of 50 × 50 μm size, at 41 retinal locations, from 50 subjects we demonstrate that CNN slightly outperforms LAPE in image quality assessment. CNN achieves accuracy of 89%, whereas LAPE metric achieves 73% and 80% (for a linear regression and random forest multiclass classifier methods, respectively) compared to ground truth. Furthermore, the retinal location, age and disease are factors that can influence the likelihood of poor image quality.

## Introduction

Adaptive optics flood illumination ophthalmoscopy (AO-FIO) allows imaging of the cone photoreceptor cells in the living human retina. However, clinical interpretation of the AO-FIO images remains challenging due to limited resolution of the systems and varied quality of images obtained. For example, the rtx1 AO flood illumination camera (AO-FIO, Imaging Eye, Orsay, France), the only commercially available instrument, is unable to resolve rod or cone photoreceptor outer segments within 2° from the center of the fovea. Moreover, as a result of the Stiles-Crawford effect, large variation in cone reflex intensity in the AO images is commonly observed, which also impacts on the quality of the information provided by the system^1, 2^. In addition, AO-FIO image quality can be reduced by defocus and the presence of cellular debris or other cell types (e.g., retinal pigment epithelium cells) in the photoreceptor layer due to acceptance of out-of-focus back-reflected light that reduces signal to noise ratio^3, 4^. Therefore, caution in AO-FIO image interpretation is required and further improvement in our assessment of image quality measurement is essential to support clinical measurements.

The challenge in image interpretation, distinguishing between good and poor image quality, and more importantly, the type and severity of the disease, is common to all ophthalmic imaging modalities. Significant progress has been made in establishing protocols for image analysis by reading centers. Traditionally, a trained reader would first grade the quality of the image and then search for specific signs of retinal pathology following a set of standardized protocol for detecting and grading disease activity^5^. However, with the increasing number of retinal imaging modalities, prevalence of retinal disease and number of clinical trials in retina, there is also a concurrent increase in demand for retinal image analysis by expert human graders^6^. Thus, there is a clinical unmet need for a more efficient and cost-effective image grading process. One solution is to develop automatic or semi-automatic methods to support image grading in population screening, routine clinical care and clinical trials. These methods for retinal image quality assessment can be based on ‘standard’ image analysis methods such as convolution of the template histogram with the image histogram computing a quality index, or more recently deep learning algorithms^7–9^. Although deep learning methods have been applied to images of cone photoreceptors for the automatic segmentation of the cone patterns, including in healthy^10, 11^ and disease^12^ images, the application of this algorithm to automatically assess image quality has not previously been explored.

In our previous work, we had investigated the use of focus measure operators to quantify AO-FIO image quality by measuring focus/defocus value of the image^13^. We had tested fifteen operators on data acquired at different focal depths and different retinal locations from healthy volunteers. The outcome of that study demonstrated differences in focus measure operator performance in quantifying AO-FIO image quality and led us to choose the energy of Laplacian (LAPE) operator as an optimal quality indicator for this imaging modality. However, the lack of a standard reference of image quality made it impossible for us to assess how good the LAPE operator was compared to ground truth of image quality classification and how its threshold should be set to separate adequate from inadequate image quality.

In this paper, detailed analysis of LAPE is undertaken to quantitatively assess its performance in evaluating AO-FIO image quality against ground truth. Owing to its relatively simple implementation, the LAPE is an attractive candidate for automated image quality assessment as it could be easily incorporated into real-time AO-FIO image processing. In this work, we also introduce and test a deep learning algorithm, called a convolutional neural network (CNN), that is commonly used as an effective tool for image classification. Both methods are compared to the ground truth established by manual graders.

Moreover, we investigate the relationship between image quality and patient-, or eye related- parameters, such as: subject age, axial length, refractive error, disease status, fixation stability and retinal location, to better understand which of these factors may influence the quality of AO-FIO images.

## Methods

### Study subjects

All research procedures described in this work followed the tenets of the Declaration of Helsinki. The research protocol was approved by Human Ethics, Office of Research Enterprise, The University of Western Australia (RA/4/1/7662, RA/4/ 1/7226, RA/4/1/5455 and RA/4/1/7457). In this retrospective study, AO-FIO images from the Lions Eye Institute (Perth, Australia) retinal camera image database were examined. Images from 28 healthy subjects (healthy controls), 11 patients with distortion and scotoma from previous macular surgery (DSM group) and 11 patients with retinal toxicity from hydroxychloroquine (HCQ group) were used for analysis. Subject demographics are summarized in the Table 1. Clinical records were reviewed for retinal diagnosis as determined by a retinal specialist (FKC).

**Table 1:** Demographic characteristic of eligible subjects.

Subjects	No of patients	No of images	Mean Age (SD) [years]	Mean axial length (SD) [mm]	Visual acuity (SD) [letter score]	Refractive error (SD) [Dioptres]	Fixation stability^†^ (SD) [degree^2^]
Healthy	28	5,740	55 (17)	24.0 (0.9)	90.1 (4.8)	0.0 (1.8)	1.1 (1.3)
DSM	11	2,255	62 (18)	23.3 (1.0)	82.8 (8.3)	− 0.5 (1.1)	1.2 (1.3)
HCQ	11	2,255	59 (7)	23.1 (0.8)	87.2 (4.9)	0.9 (1.8)	2.9 (4.2)

^†^Bivariate contour ellipse area, 63% derived from MAIA microperimetry.

DSM; distortion and scotoma from macular surgery group, HCQ; hydroxychloroquine toxicity group, SD; standard deviation.

Inclusion criteria for all subjects included absence of media opacity, spherical refractive errors of less than − 6 diopters (D) of myopia, less than + 4 D of hyperopia, and ability to give informed consent. Eyes included in the healthy group had best-corrected visual acuity of greater than 80 letters on the Early Treatment Diabetic Retinopathy Study (ETDRS) chart. Subjects in the DSM group had distortion and/or scotoma elucidated on Amsler grid testing. HCQ toxicity diagnosis was based on a history of HCQ intake, pericentral visual field defect, and typical optical coherence tomography and multifocal electroretinography features. Axial length measured with IOL Master500 (Carl Zeiss Meditec, Dublin, CA, USA), refractive error measured with autorefractor (Ark1, Auto Ref/Keratometer; Nidek, Gamagori, Japan) and fixation stability measured with microperimetry (MAIA, Centervue, Padova, Italy) were also recorded.

### Adaptive optics retinal camera

Retinal photoreceptor images were acquired using flood illumination adaptive optics ophthalmoscopy (AO-FIO) (rtx1, Imagine Eyes, Orsay, France). Each AO-FIO image acquisition consists of 40 consecutive AO-FIO frames recorded over 4 s. The visibility of the cone photoreceptors reflex is improved by the increased signal-to-noise ratio of the AO-FIO image reconstructed from co-registration of individual AO-FIO frames using a cross-correlation method (registration of X/Y translation and rotation) and averaging. The raw images, which show artefacts due to eye blinking and saccades, are automatically eliminated by the acquisition software during this registration process. Unless specified, this post-processed AO image (after alignment and averaging) is the one used for further analysis in this report. The final AO image corresponds to a square region of the retina traversing 4° × 4° of visual field (750 × 750 pixels, oversampled to 1500 × 1500 pixels). On the retina, the linear dimensions are approximately 1.2 × 1.2 mm. The resolution of the system limits the ability to distinguish cone photoreceptor structures that are 2 µm or less^14^. Therefore, the images acquired from this system are not suitable for visualizing cone photoreceptor outer segments within 2° from the foveal center nor the outer segments of the rod photoreceptor cells.

### Adaptive optics image capture and processing protocol

**Figure 1:**
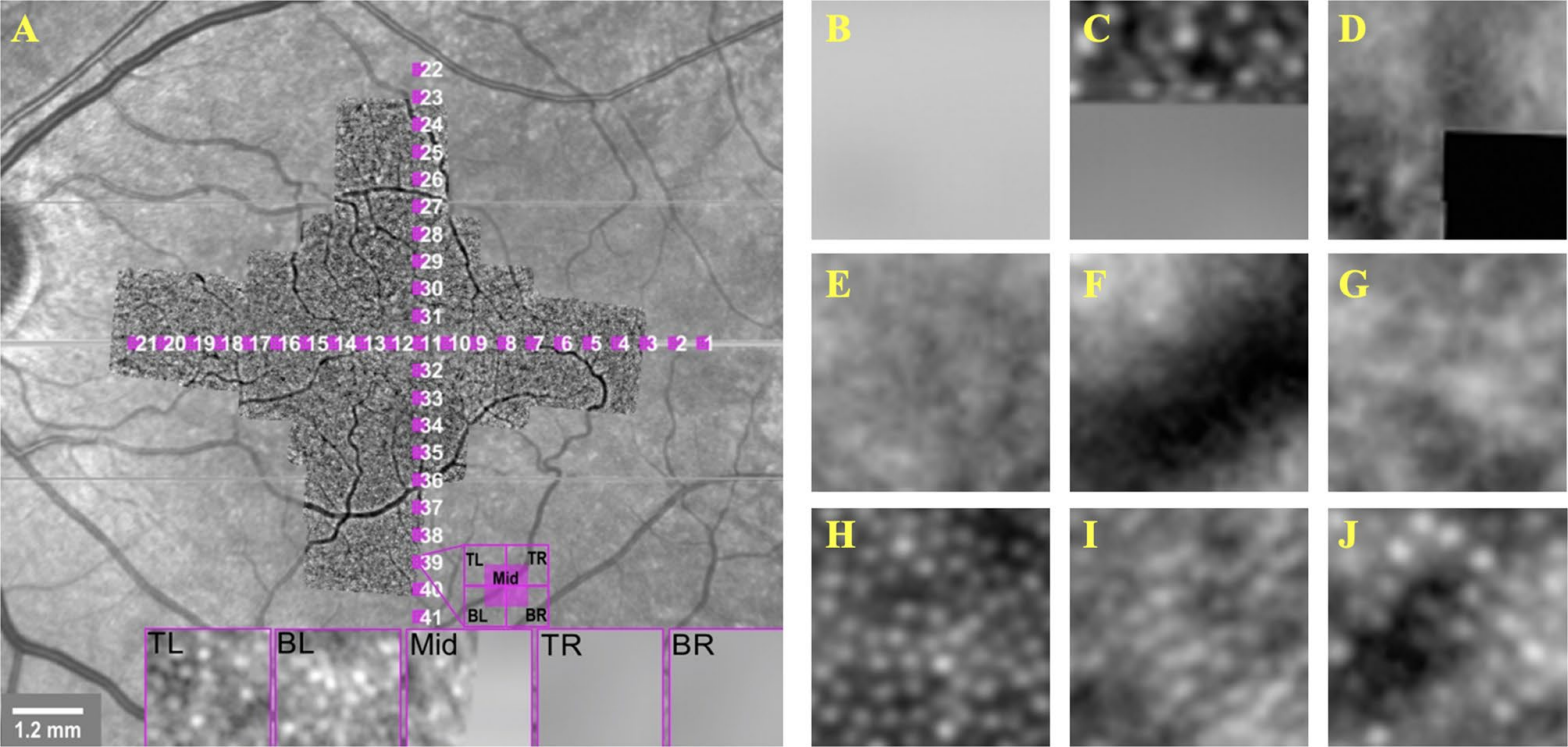
(**A**) AO-FIO mosaic overlaid on fundus SLO images with marked regions of interest (numbered purple squares). The cones are counted in five sampling windows per locus, each of 50 × 50 µm. The middle window (Mid) is located exactly at the center of the locus, and Top Left (TL), Top Right (TR), Bottom Left (BL), and Bottom Right (BR) are shifted and partially overlapped with Mid (see example at the bottom of **A**). Example with a set of representative images for each category: Category 1 (**B**–**D**), Category 2 (**E–G**), Category 3 (**H**–**J**). Images of category 1 are from the edge of AO-FIO mosaics, whereas 2 are associated with poor image quality or the presence of vessels in the AO-FIO mosaic.

A total of 20 consecutive single images with 1° to 2° of overlap were acquired from each participant. For visualization purposes, the overlapping single AO images were stitched together to reconstruct a wide-field AO montage by using the MosaicJ plugin for ImageJ (Laboratory for Optical and Computational Instrumentation, Madison, WI). The location of the foveal center in the wide-field AO montage was determined through alignment with a single horizontal spectral domain optical coherence tomography (OCT) scan and a high quality near infrared image using Adobe Photoshop CS6 (Adobe Systems, Inc., San Jose, CA). The images were corrected for magnification error related to axial length (AL) using the modified Littmann’s method described by Bennett et al.^15^, $q=0.013063\times (AL-1.82)$ where q represents the magnification factor. For more details, refer to Chew et al.^16^ Regions of interest (ROI) were chosen from retinal loci along the vertical and horizontal meridians. These ROIs were spaced 1° apart between 1° and 10° from the foveal center along each meridian (Fig. 1A). Each ROI was divided into five overlapping sampling windows measuring 50 × 50 µm (approx. 65 × 65 pixels). These were extracted using custom software.

### The ground truth reference standard dataset

AO-FIO images were classified into three categories (Fig. 1 B-J). These were defined as follows: category 1—more than 5% of the image truncated category 2—less than 5% truncation but the remaining image had no visible/resolvable cones in at least one quadrant of the image; category 3—less than 5% truncation with the remaining image showing visible cones in all quadrants of the image. Those images in categories 1 and 2 are considered as unacceptable for clinical use or image analysis due to missing features in the image (category 1) and poor image quality (category 2), whilst category 3 are considered as acceptable for quantitative analysis. Image category 1 is introduced to prevent the analysis of images at the edge of the mosaics, which could happen if ROI is selected and cropped automatically from the series of mosaics of different sizes.

All AO-FIO images (10,250 images from 50 participants, Table 1) were graded by three graders (DMS—expert, four-year experience with AO-FIO data; JL—intermediate, two-year experience; DR—beginner, 6 months experience) using the categories defined above. Each grader was given a short tutorial on the grading system reinforced by a range of illustrative cases (i.e. example images) for all three categories. These cases were excluded from analyzed dataset. Each grader performed the assessment independently using a custom graphic user interface, developed in Matlab (MathWorks, Massachusetts, United States). Images were displayed in a sequential randomized order, and the grader had to select the corresponding category buttons (1, 2 or 3) to confirm the grade. Graders were masked to focus operator value, biometric data, subject condition (health/disease, age), image location in the retina and the results of the other graders.

The ground truth reference grade (the “ground truth”) used for the validation of automatic image quality assessment methods was based on the consensus from real-time discussion between the three graders in cases of disagreement between two or three graders. The expert grader facilitated discussion and was trying to give opinion as the last one to not influence less-experienced graders. We used the process of adjudication described by Krause et al. to reduce manual grading errors^17^.

### Manual graders’ performance

All images (10,250 images from 50 participants, Table 1) were used to investigate the distribution of images per category amongst different graders. The agreement between graders prior to consensus and a reference standard was investigated using a subset of images (5535 images from 27 participants, Supp. Table 3). This is the same dataset used to evaluate the performance of LAPE and CNN against a reference standard. This ensures a fair comparison while assessing the agreement between graders, LAPE and CNN performance on exactly the same dataset to avoid any bias.

### Energy of Laplacian focus operator performance

**Figure 2:**
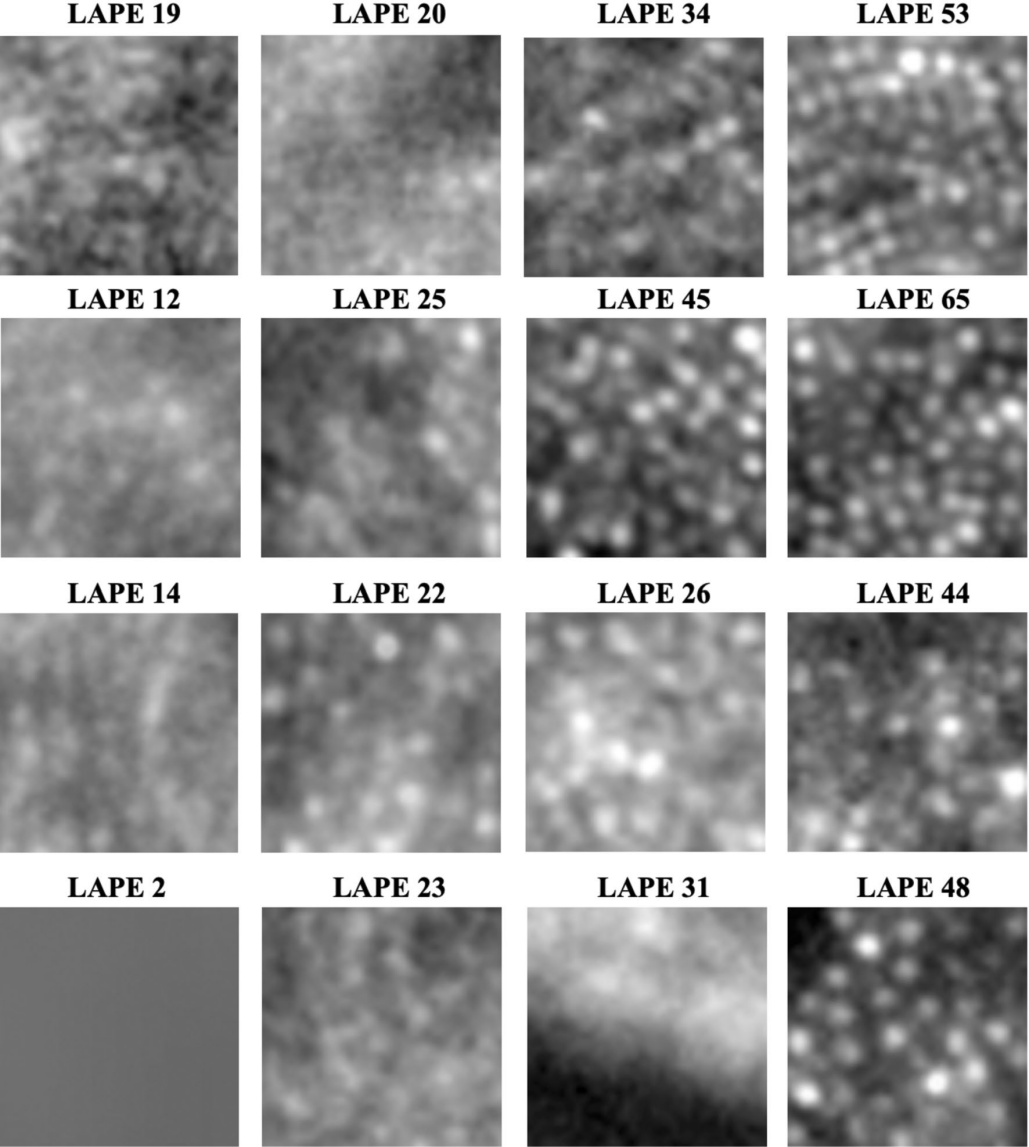
AO-FIO images and their LAPE values. Each row shows images from 2, 4, 6, and 8 degrees from the fovea respectively. Each image is 50 × 50 µm size.

The energy of Laplacian focus operator (LAPE) enables to quantify image quality by measuring focus/defocus value of the image. Briefly, the input image is first normalized for brightness and later convolved with a Laplacian kernel. After, the energy (sum of squared intensity values) of the characterized image is computed and single number per image is extracted^18^. In case of ours AO-FIO images the LAPE focus value ranges from 0 to 75. The global LAPE was measured for each 50 × 50 µm image (10,250 images from 50 participants, Table 1). This value was used to investigate the average LAPE value for each image category and the distribution of images per image grade category amongst different LAPE groups. Figure 2 shows examples of images and LAPE values associated with each of them.

Furthermore, to be able to validate the LAPE performance against a ground truth and the CNN methods, we applied and compared two different multiclass classifier algorithms; these help to identify to which of the three image categories (1,2,3) a LAPE value (0–75) belongs. First, a method based on linear regression was applied. The linear regression model aims to predict a value based on a linear combination of inputs; in our case, only a single input feature is given so the value is based on a linear scaling of the input. This value is then rounded to 1, 2, or 3, giving the class of the input. We also used a random forest classifier with 1,000 trees in the “classification” mode ^19^ (with the “ranger” engine)^20^. The random forest is a popular machine learning algorithm that, for a set of predictors, will predict the probability of falling into each of the classifications. It prepares the probability curves by predicting image classification over a sequential range of LAPE scores from 0 to 75 in 0.1-unit increments and applying Loess smoothing in the ggplot2 R package^21^. Estimated LAPE thresholds are set at the intersection of the probability curves, noting that at the 50% point, the model reports equal probability of being in a category and is classified into each of the 3-image categories.

Both models were trained on 4715 images from 23 patients, a subset of the 50 patients, (Supp. Table 1) to maximize overall accuracy and then validated using the remaining 5535 images from 27 participants (Supp. Table 3). LAPE thresholds to predict cut-offs for each of the image classifications were estimated using the Tidymodels collection of packages in R^22^. Model performance was assessed with tenfold cross-validation on the training data^23^ and reported through confusion matrices and accuracy of the methods—the percentage of how often the classifier was correct. Estimated LAPE thresholds are set at the intersection of the probability curves between image categories (*i.e.* image classification was made when the associated probability of assignment to an image category was greater than 50%).

### Convolutional neural network performance

A convolutional neural network (CNN) is a form of neural network often used for a range of image analysis tasks, including image classification. CNNs are built of many layers, normally arranged in a particular order (convolution → activation → subsampling blocks), which progressively builds a larger model of the image as a whole. Within the convolutional layers, neural networks learn a number of “filters”, each based on linear combinations of the input to extract a particular image feature. These filters are tuned to create the correct output for a given input.

To suit the network size, the AO-FIO images were trimmed to the central 65 × 65 pixels if larger or mirrored if smaller^24^. The network used was a CIFAR network modified to accept a 65 × 65 sample. Similar architectures have been applied to OCT boundary classification^24^. The network architecture is shown in Supp. Figure 1. The CNN network was trained on a dataset as summarized in Supp. Table 1. Also, a small part of this training dataset was used to validate the model, which facilitates assessment of how well the network is trained and ensures the learning is generalizing well. The network was then evaluated on the validation dataset presented in Supp. Table 3. This is the same dataset used to evaluate the performance of manual graders and LAPE against a reference standard.

### Factors affecting image quality

We use logistic regression on the data set in Table 1 to ascertain the impact of subject age, axial length, refractive error, fixation stability, disease status and location in the retina on the likelihood of poor image quality (category 2). We didn’t include images from category 1 into the analysis since they represent images with insufficient features.

### Statistical analysis

Subject demographics were summarized by their mean and standard deviation (SD). Cohen’s kappa (κ) statistic was used to calculate agreement between graders^25^. The proportions of acceptable and unacceptable images, based on adjudicated manual grading scores and LAPE values are presented separately for subjects in each study group: healthy, DSM and HCQ. To evaluate the ability of LAPE to differentiate different manual grades, the mean and median of LAPE for each of the 3 manual grading groups were calculated and compared using the Kruskal–Wallis test. Efficiency of the manual grading, LAPE and CNN were measured against a reference standard and displayed by a confusion matrix. The accuracy of each method is defined by the percentage of how often the classifier was correct.

## Results

### Manual graders performance

**Figure 3:**
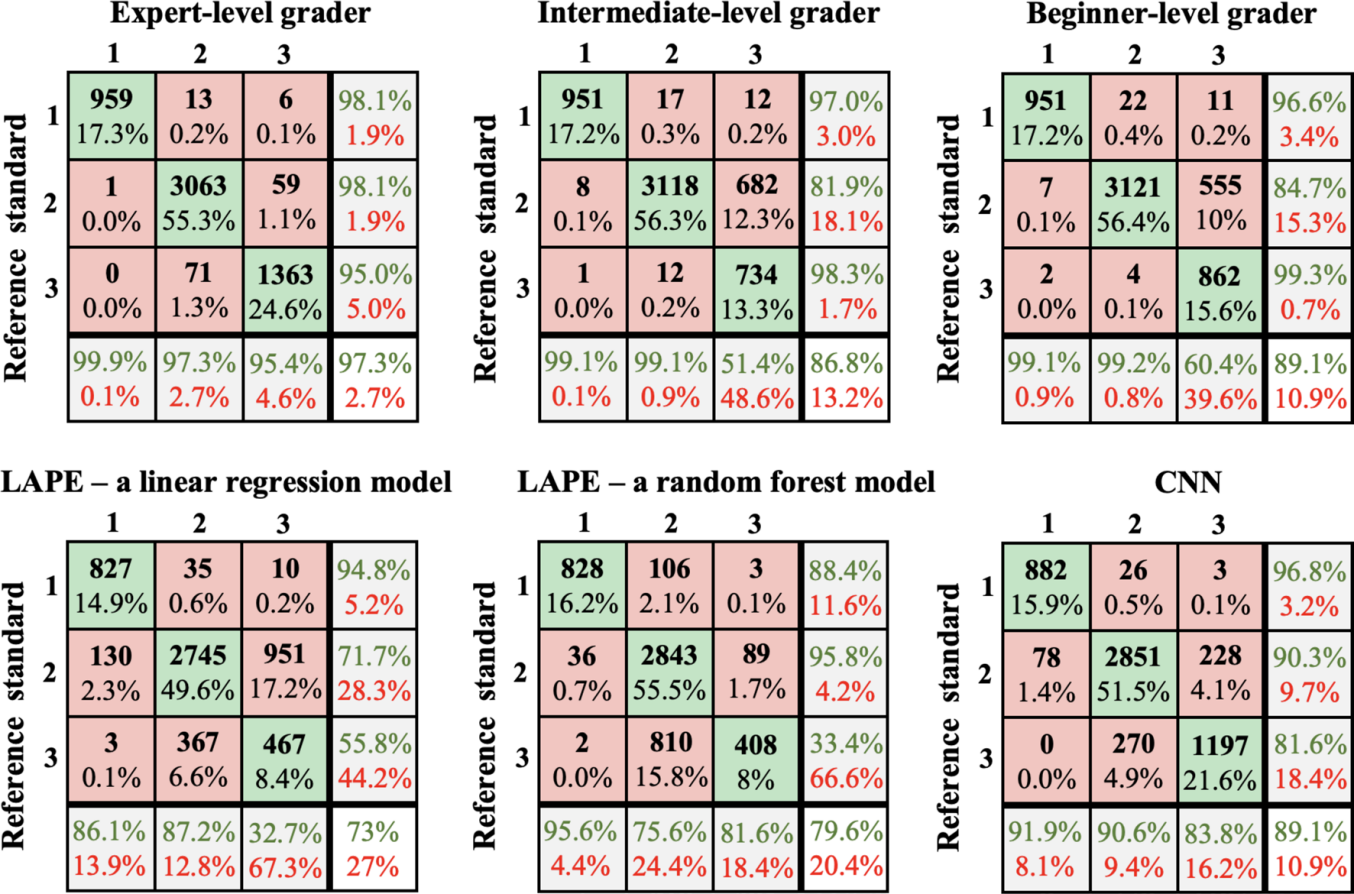
Confusion metrices showing the performance of LAPE, CNN and manual graders against a reference standard. Accuracy of the method is defined by percentage of how often the classifier was correct (green values).

Overall, there was substantial agreement between each pair of graders, as well as between each grader and the reference standard scores (κ equal or greater than 0.673) for all three study groups. The highest agreement was observed in DSM group images (κ equal or greater than 0.883). The results are summarized in Supp. Table 4. While considering the combined three groups, 85% of the manual quality grading had complete agreement between the 3 graders. The highest agreement was in defining category 1 images (Suppl. Table 5). Following adjudication of images with grading discrepancy there were 2,052 (20%), 5,728 (56%), and 2,470 (24%) images assigned the “ground truth” categories of 1, 2 and 3 respectively (Supp. Table 5). As presented in Fig. 3, the three graders, 1—an expert, 2—an intermediate, and 3—a beginner, achieved overall accuracies of 97%, 87% and 89% respectively, compared to the “ground truth”.

### LAPE performance

LAPE values increased with improving manual-graded image category in all three study groups (from category 1 to 3, Kruskal–Wallis test, *p* < 0.001, Supp. Table 6). However, the range of LAPE values overlapped considerably between manual grading groups, in particular for categories 2 and 3 (Supp. Tables: 6, 7).

When tested against the ground truth, LAPE linear regression model achieved an overall accuracy of 73% as demonstrated by the confusion metric (Fig. 3). LAPE ranges for each category were found to be for category 1: < 8; category 2: between 8 and 35; category 3: LAPE > 35.

The LAPE random forest model against the ground truth achieved an overall accuracy of 80% as demonstrated by the confusion metric (Fig. 3). LAPE ranges for each category were found to be for category 1: < 7; category 2: between 7 and 38; category 3: LAPE > 38.

### CNN performance

The CNN performed better than the LAPE linear regression model, with better values through the entire confusion matrix (Fig. 3). The overall accuracy of the CNN was 89%. Like the regression model, the majority of error results from confusion between categories 2 and 3.

### Factors affecting image quality

**Figure 4:**
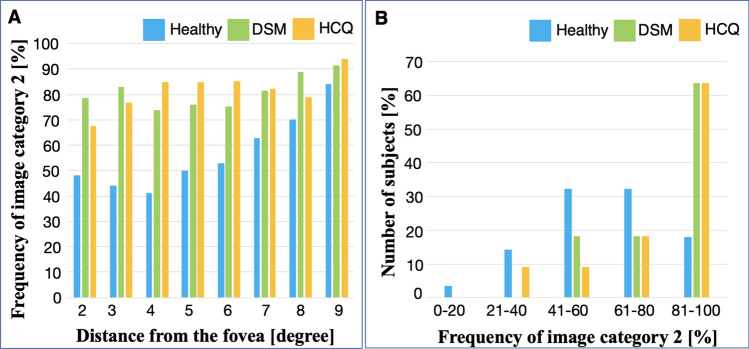
(**A**) Frequency of image category 2 (based on ground truth) at each retinal location in healthy subjects (blue), patients with distortion and scotoma from macular surgery (DSM group, green) and hydroxychloroquine toxicity (HCQ group, orange). Images more than 10° from foveal centre were excluded from the analysis due to the low number of images available for grading. (**B**) Proportion of subjects assigned image category 2.

Logistic regression suggested that images of patients with retinal disease were 5.2 times more likely to be categorized as bad image quality (image category 2) than those from healthy subjects (OR of healthy vs diseased of 0.19, 95% CI: 0.09 to 0.43), keeping other factors consistent. For every increased degree distance from the fovea, the odds of obtaining a poor-quality image increased by an odds ratio of 1.03 (95% CI: 1.01 to 1.03). The odds ratio of a patient producing poor quality image increased by a ratio of 1.03 (95% CI: 1.00 to 1.06) for each year increase in age. There was no evidence to suggest an association between axial length (OR: 1.16, 95% CI: 0.73 to 1.83), spherical equivalent (OR: 1.10, 95% CI: 0.82 to 1.46), best corrected visual acuity (OR: 0.98, 95% CI: 0.92 to 1.05), and fixation stability given by parameter BCEA63 (OR: 1.03; 95% CI: 0.81 to 1.31). As demonstrated in Fig. 4A, the frequency of images graded as category 2 is high and is similar for all retinal locations, with higher frequencies of good quality images occurring at retinal loci between 2° and 6° from the foveal centre. Figure 4B demonstrates the frequency of image category 2 per subject. Approximately 80% healthy subjects and 90% of patients with the retinal disease had half or more images category 2, in the group of images category 2 and 3. Images category 1 were excluded from this analysis.

## Discussion and conclusions

The development of automated grading systems for AO-FIO image quality are needed to facilitate accurate large volume image-grading of AO-FIO images. The result in this report is based on a robust ground truth grading method as demonstrated by the substantial inter-grader agreement in our simple 3-category manual grading system.

CNN performed well when compared with manual grading results, achieving an overall classification accuracy of 89% when tested on a dataset of 5,535 images. Previously published reports on using CNN for classification of normal or mild vs moderate or severe diabetic retinopathy have achieved accuracies in the range of 90%, using large data sets of 80,000 to 120,000 images^26, 27^. Lam et al. introduced an automatic diabetic retinopathy grading system capable of classifying images based on disease pathologies from four severity levels. Their algorithm achieved 95% evaluation accuracy using 35,000 images with 5 class labels (normal, mild, moderate, severe, end stage)^9^. The performance of deep learning methods, like the one used in this study, is known to improve with increasing number of images. Therefore, the performance of the proposed CNN can be improved further with increasing the size of AO-FIO datasets.

We could not find any report that describes the use of the focus operator (LAPE) as an automated method for image grading. Our results showed that the focus operator applied to 5,535 images obtained a classification performance of 73% (linear regression classification model) and 80% (random forest classifier), which indicates that precision of performance can be improved with the choice of a more complex algorithm to solve the classification problem. However, in both cases obtained results are less accurate than the CNN.

The cause of poor image quality in AO-FIO images is mostly associated with the presence of retinal disease, retinal location and age. The quality of AO-FIO images can be degraded by defocus. Further degradation of image quality can occur in diseased eye when defocused light is reflected back from cellular debris or other cells types (e.g. retinal pigment epithelial cells) in the outer retinal layers, thus reducing signal to noise ratio. It is also the case that although photoreceptor cells may appear structurally intact, a loss of function of these cells may impair its wave-guiding properties and this can have a deleterious impact on image sharpness^28^. Other factors thought to influence the reflectivity of the photoreceptor outer segments include time of day, natural physiological variation within cells, photocurrent dynamics inducing refractive index changes, and the coherence properties of the projected light^29–31^. Additionally, rapid eye movement and wavefront aberrations that have been unaccounted for in the device may also contribute to reductions in image quality, hence presenting significant challenges to the clinical interpretation of AO-FIO images^32, 33^. For example, Chew et al. have reported exclusion of approximately 40% of images due to image quality in their analyzed data set^16^. Debellemaniere et al. had to exclude at least one eye from 46.9% of subjects due to poor AO image quality in their study of patients taking hydroxychloroquine without any evidence of maculopathy^34^. Furthermore, Feng et al. reported that in their study of AO-FIO images of healthy subjects aged 14 to 69, the images from 52.7% were excluded due to image quality^35^. We showed a similar frequency of images belonging to categories 2 (unacceptable for evaluation), being 50–60% at retinal loci spanning 2° to 6° from the foveal center. Image rejection rate was even higher for retinal loci at 7° and 8° (75%), 9° (95%) from the foveal center (Fig. 3). Further investigation will be required into the robustness of the methods with regard to the number of valid images required in order to make a valid classification.

There are a number of limitations in our study. Images taken for the analysis had only a narrow range of retinal disease diagnoses and the imaging was performed by clinicians with varying levels of experience in operating the AO-FIO instrument. Therefore, further evaluation is required in a larger and more diverse cohort of patients. We didn’t measure the intra-individual variation of our manual grading and we didn’t investigate its impact on the quantitative AO-FIO measurements and their repeatability.

The proposed AO-FIO image automated grading system could be incorporated into AO-FIO image acquisition software to determine image quality and inform users when acquisition should be repeated or the image interpreted with caution. Similar solutions have already been applied in commercial optical coherence tomography/angiography devices and have been well received.

The current limitation of the proposed grading system is its inability to categorize causes of impaired cone visualization; it is unknown whether the poor image quality is due to retinal disease, imaging artifact, or perhaps a combination of both. Retinal pathology is an unlikely explanation in our cohort since patients in both DSM and HCQ groups had only mild structural damage and complete loss of cone photoreceptor cells was not expected. However, further studies are required to distinguish between cone loss due to retinal pathology and inability to resolve cones due to poor image quality. This might increase the applicability of the modality to the evaluation of a range of retinal pathologies. Nevertheless, our proposed methodology at least allows us to reduce the number of images that should be revised by a clinician or researcher to decide if one should proceed with qualitative or quantitative analysis. Following Gale et al.’s recommendation we also suggest correlation between AO-FIO images and other retinal imaging modalities, including optical coherence tomography and fundus autofluorescence, to increase confidence in the AO-FIO results.3,4 AO-FIO is a powerful imaging modality, which also presents its own challenges in data interpretation. Further improvement in automatic image grading methods has the potential to increase the utility of this imaging modality in clinical practice.

In summary, we have demonstrated that our CNN classifier outperforms a defocus measurement classifier using the LAPE values (random forest multiclass classifier method and a linear regression) in AO-FIO image quality assessment (accuracy: 89% vs 84% vs 73%). However, further improvement of the algorithm and/or increase in size of the training dataset should be undertaken to achieve a better performance to match results obtained by expert level grader (accuracy: 97%). Retinal location and age are the only parameters that impact on the frequency of poor-quality images. We recommend limiting the collection of AO-FIO images to retinal loci between 2° and 6° from the foveal center to maximize the frequency of obtaining analyzable images.

## Supplementary Information

Supplementary Information.

## Data Availability

All relevant data are within the paper. The CNN/LAPE algorithm can be available upon request.

## References

[1] Westheimer G (2008). Directional sensitivity of the retina: 75 years of Stiles-Crawford effect. Proc. R. Soc. B: Biol. Sci..

[2] Mariotti L, Devaney N, Lombardo G, Lombardo M (2016). Understanding the changes of cone reflectance in adaptive optics flood illumination retinal images over three years. Biomed. Opt. Express.

[3] Gale MJ, Harman GA, Chen J, Pennesi ME (2019). Repeatability of adaptive optics automated cone measurements in subjects with retinitis pigmentosa and novel metrics for assessment of image quality. Transl. Vis. Sci. Technol..

[4] Gale MJ, Feng S, Titus HE, Smith TB, Pennesi ME (2016). Interpretation of flood-illuminated adaptive optics in subjects with retinitis pigmentosa. Adv. Exp. Med. Biol..

[5] Wu L, Fernandez-Loaiza P, Sauma J, Hernandez-Bogantes E, Masis M (2013). Classification of diabetic retinopathy and diabetic macular edema. World J. Diabetes.

[6] Alipour SHM, Rabbani H, Akhlaghi MR (2012). Diabetic retinopathy grading by digital curvelet transform. Comput. Math. Methods Med..

[7] Hunter A, Lowell JA, Habib M, Ryder B, Basu A, Steel D (2011). An automated retinal image quality grading algorithm. Proc. Ann. Int. Conf. IEEE Eng. Med. Biol. Soc..

[8] Tufail A (2016). An observational study to assess if automated diabetic retinopathy image assessment software can replace one or more steps of manual imaging grading and to determine their cost-effectiveness. Health Technol. Assess..

[9] Lam C, Yi D, Guo M, Lindsey T (2018). Automated detection of diabetic retinopathy using deep learning. AMIA Summits Transl. Sci. Proc..

[10] Cunefare D, Fang L, Cooper RF, Dubra A, Carroll J, Farsiu S (2017). Open source software for automatic detection of cone photoreceptors in adaptive optics ophthalmoscopy using convolutional neural networks. Sci. Rep..

[11] Hamwood J, Alonso-Caneiro D, Sampson D, Collins MJ, Chen FK (2019). Automatic detection of cone photoreceptors with fully convolutional networks. Transl. Vis. Sci. Technol..

[12] Davidson B, Kalitzeos A, Carroll J, Dubra A, Ourselin S, Michaelides M, Bergeles C (2018). Automatic cone photoreceptor localisation in healthy and Stargardt afflicted retinas using deep learning. Sci. Rep..

[13] Alonso-Caneiro D, Sampson DM, Chew AL, Collins MJ, Chen FK (2018). Use of focus measure operators for characterization of flood illumination adaptive optics ophthalmoscopy image quality. Biomed. Opt. Express.

[14] Muthiah MN (2014). Cone photoreceptor definition on adaptive optics retinal imaging. Br. J. Ophthalmol..

[15] Bennett AG, Rudnicka AR, Edgar DF (1994). Improvements on Littmann method of determining the size of retinal features by fundus photography. Graefes Arch. Clin. Exp. Ophthalmol..

[16] Chew AL, Sampson DM, Kashani I, Chen FK (2017). Agreement in cone density derived from gaze-directed single images versus wide-field montage using adaptive optics flood illumination ophthalmoscopy. Transl. Vis. Sci. Technol..

[17] Krause J (2018). Grader variability and the importance of reference standards for evaluating machine learning models for diabetic retinopathy. Ophthalmology.

[18] Pertuz S, Puig P, Garcia MA (2013). Analysis of focus measure operators for shape-from-focus. Pattern Recognit..

[19] Kuhn, M. and Vaughan, D. *Parsnip: A common API to modeling and analysis functions*. https://CRAN.R-project.org/package=parsnip (2020).

[20] Wright, M. N., Wager, S. and Probst, P. *Ranger: A fast implementation of random forests*. https://CRAN.R-project.org/package=ranger (2020).

[21] Wickham, H., Chang, W., Henry, L., Pedersen, T.L., Takahashi, K., Wilke, C., Woo, K., Yutani, H. and Dunnington, D. *Ggplot2: Create elegant data visualisations using the grammar of graphics*. https://CRAN.R-project.org/package=ggplot2 (2020).

[22] Kuhn, M. and Wickham, H. *Tidymodels: Easily install and load the ‘Tidymodels’ packages*. https://CRAN.R-project.org/package=tidymodels (2020).

[23] Kuhn, M., Chow, F., and Wickham, H. *Rsample: General resampling infrastructure*. https://CRAN.R-project.org/package=rsample (2020).

[24] Hamwood J, Alonso-Caneiro D, Read SA, Vincent SJ, Collins MJ (2018). Effect of patch size and network architecture on a convolutional neural network approach for automatic segmentation of OCT retinal layers. Biomed. Opt. Express.

[25] McHugh ML (2012). Interrater reliability: The kappa statistic. Biochem. Med..

[26] Gargeya R, Leng T (2017). Automated identification of diabetic retinopathy using deep learning. Ophthalmology.

[27] Gulshan V (2016). Development and validation of a deep learning algorithm for detection of diabetic retinopathy in retinal fundus photography: Accuracy of a deep learning algorithm for detection of diabetic retinopathy. JAMA.

[28] Merino D, Duncan JL, Tiruveedhula P, Roorda A (2011). Observation of cone and rod photoreceptors in normal subjects and patients using a new generation adaptive optics scanning laser ophthalmoscope. Biomed. Opt. Express.

[29] Pallikaris A, Williams DR, Hofer H (2003). The reflectance of single cones in the living human eye. Investig. Ophthalmol. Vis. Sci..

[30] Bruce KS (2015). Normal perceptual sensitivity arising from weakly reflective cone photoreceptorsnormal perceptual sensitivity of weakly reflective cones. Invest. Ophthalmol. Vis. Sci..

[31] Kocaoglu OP (2016). Photoreceptor disc shedding in the living human eye. Biomed. Opt. Express.

[32] Lombardo M, Serrao S, Ducoli P, Lombardo G (2013). Influence of sampling window size and orientation on parafoveal cone packing density. Biomed. Opt. Express.

[33] Bidaut Garnier M (2014). Reliability of cone counts using an adaptive optics retinal camera. Clin. Exp. Ophthalmol..

[34] Debellemanière G (2015). Assessment of parafoveal cone density in patients taking hydroxychloroquine in the absence of clinically documented retinal toxicity. Acta Ophthalmol..

[35] Feng S (2015). Assessment of different sampling methods for measuring and representing macular cone density using flood-illuminated adaptive optics. Investig. Ophthalmol. Vis. Sci..

