# Supplementary material for: Evaluation of focus and deep learning methods for automated image grading and factors influencing image quality in adaptive optics ophthalmoscopy
Source: Sci Rep. 2021 Aug 17;11:16641. doi: 10.1038/s41598-021-96068-2 (PMC8371000; doi:10.1038/s41598-021-96068-2)
Supplement: Supplementary file 1 — Supplementary Information. [file 41598_2021_96068_MOESM1_ESM.docx]

**Evaluation of focus and deep learning methods for automated image grading and factors influencing image quality in adaptive optics ophthalmoscopy**

Danuta M Sampson^1,2^, David Alonso-Caneiro^1,3^, Avenell L Chew^1^, Jonathan La^1^, Danial Roshandel^1^, Wang Yufei^4^, Jane Khan^5,6^, Enid Chelva^6^, Paul G. Stevenson,^7^ Fred K Chen^1,5,8,*^

**Supplementary Table 1** Demographic characteristic of subject subset 1, chosen from the group of 50 available subjects, for training LAPE random forest classification model and CNN.

| Subjects | No of patients | No of images | Age | Axial length [mm] | Visual Acuity | Spherical Equivalent [D] | Fixation Stability |
| --- | --- | --- | --- | --- | --- | --- | --- |
| Healthy | 11 | 2,255 | 55 (19) | 23.9 (0.9) | 88.3 (5.1) | -0.6 (2.3) | 1.5 (1.8) |
| DSA | 4 | 820 | 69 (7) | 23.3 (0.7) | 78 (11.6) | -0.8 (0.4) | 1.5 (1.8) |
| HCQ | 4 | 820 | 60 (5) | 23.5 (0.5) | 86.3 (2.1) | +0.1 (1.4) | 1.7 (1.8) |

**Supplementary Table 2** Demographic characteristic of subject subset 2, chosen from the group of 50 available subjects, for validation of LAPE random forest classification model and CNN.

| Subjects | No of patients | No of images | Age | Axial length [mm] | Visual Acuity | Spherical Equivalent [D] | Fixation Stability |
| --- | --- | --- | --- | --- | --- | --- | --- |
| Healthy | 2 | 410 | 56 (3) | 24.2 (0.8) | 90 (5.7) | +0.7 (0.3) | 0.5 (0.1) |
| DSA | 1 | 205 | 77 | 21.5 | 85 | +0.8 | 0.4 |
| HCQ | 1 | 205 | 53 | 23.6 | 96 | -0.3 | 0.5 |

**Supplementary Table 3** Demographic characteristic of subject subset 3, remained after choosing data for training and validation. This dataset is used for evaluation and comparison between LAPE random forest classification model, CNN and manual graders against a reference standard.

| Subjects | No of patients | No of images | Age | Axial length [mm] | Visual Acuity | Spherical Equivalent [D] | Fixation Stability |
| --- | --- | --- | --- | --- | --- | --- | --- |
| Healthy | 15 | 3,075 | 52 (19) | 23.9 (0.9) | 88.5 (4.8) | -0.4 (2.0) | 1.4 (1.6) |
| DSA | 6 | 1,230 | 55 (20) | 23.6 (0.9) | 85.7 (4.7) | -0.4 (1.3) | 1.2 (1.0) |
| HCQ | 6 | 1,230 | 59 (8) | 22.7 (0.8) | 86.3 (4.9) | +1.6 (1.8) | 4.0 (5.0) |

**Supplementary Table 4** Agreement between graders as well as graders and reference standard in AO-FIO image assessments of Healthy, DSA and HCQ study groups separately; precision indicates how often graders agreed.

|  | **Healthy subjects** | | **DSM** | | **HCQ** | |
| --- | --- | --- | --- | --- | --- | --- |
| **Grader** | Cohen’s kappa coefficient (κ) | Precision [%] | Cohen’s kappa coefficient (κ) | Precision [%] | Cohen’s kappa coefficient (κ) | Precision [%] |
| **1-2** | 0.673 | 79.0 | 0.883 | 93.7 | 0.784 | 91.7 |
| **1-3** | 0.761 | 84.7 | 0.894 | 94.3 | 0.776 | 91.4 |
| **2-3** | 0.796 | 87.9 | 0.891 | 94.4 | 0.868 | 95.6 |
| **1-RS** | 0.915 | 94.5 | 0.967 | 98.2 | 0.961 | 98.4 |
| **2-RS** | 0.724 | 82.7 | 0.897 | 94.5 | 0.810 | 92.8 |
| **3-RS** | 0.827 | 89.1 | 0.902 | 94.7 | 0.795 | 92.2 |

1 – expert-level grader, 2 – intermediate-level grader, 3 – beginner-level grader, RS – a reference standard

**Supplementary Table 5** Distribution of images per image grade category amongst different graders.

|  | **Healthy subjects** | | | **DSM** | | | **HCQ** | | |
| --- | --- | --- | --- | --- | --- | --- | --- | --- | --- |
|  | **Category** | | | **Category** | | | **Category** | | |
| **Grader** | **1** | **2** | **3** | **1** | **2** | **3** | **1** | **2** | **3** |
| **1** | 1179 | 2638 | 1906 | 609 | 1348 | 293 | 263 | 1656 | 332 |
| **2** | 1174 | 3500 | 1038 | 598 | 1434 | 212 | 261 | 1812 | 171 |
| **3** | 1178 | 3204 | 1331 | 606 | 1428 | 210 | 259 | 1817 | 168 |
| **RS** | 1184 | 2717 | 1839 | 606 | 1339 | 310 | 262 | 1672 | 321 |

1 – expert-level grader, 2 – intermediate-level grader, 3 – beginner-level grader, RS – a reference standard

**Supplementary Table 6** Average LAPE for each image-category.

|  | **Healthy subjects** | | | **DSM** | | | **HCQ** | | |
| --- | --- | --- | --- | --- | --- | --- | --- | --- | --- |
|  | **Category** | | | **Category** | | | **Category** | | |
| **LAPE** | **1** | **2** | **3** | **1** | **2** | **3** | **1** | **2** | **3** |
| **Mean (SD)** | 3  (5) | 25  (8) | 34  (10) | 3  (4) | 21  (8) | 29  (9) | 5  (8) | 25  (11) | 32  (10) |
| **Median (range)** | 2  (0-35) | 24  (6-63) | 34  (1-73) | 2  (0-36) | 19  (7-55) | 28  (2-60) | 2  (0-43) | 25  (0-60) | 31  (11-63) |

**Supplementary Table 7** Distribution of images per image grade category amongst different LAPE groups.

|  | **Healthy subjects** | | | **DSM** | | | **HCQ** | | |
| --- | --- | --- | --- | --- | --- | --- | --- | --- | --- |
|  | **Category** | | | **Category** | | | **Category** | | |
| **LAPE** | **1** | **2** | **3** | **1** | **2** | **3** | **1** | **2** | **3** |
| **0-20** | 1154 | 903 | 100 | 596 | 739 | 51 | 248 | 612 | 40 |
| **21-30** | 22 | 1150 | 632 | 8 | 411 | 128 | 11 | 605 | 105 |
| **31-40** | 8 | 555 | 691 | 2 | 173 | 105 | 2 | 301 | 115 |
| **41-50** | 0 | 106 | 299 | 0 | 12 | 22 | 1 | 126 | 43 |
| **51-60** | 0 | 2 | 96 | 0 | 4 | 4 | 0 | 28 | 16 |
| **61-70** | 0 | 1 | 19 | 0 | 0 | 0 | 0 | 0 | 2 |
| **71-80** | 0 | 0 | 2 | 0 | 0 | 0 | 0 | 0 | 0 |
| **Total** | 1184 | 2717 | 1839 | 606 | 1339 | 310 | 262 | 1672 | 321 |
| **Total [%]** | **21** | **47** | **32** | **27** | **60** | **14** | **12** | **74** | **14** |


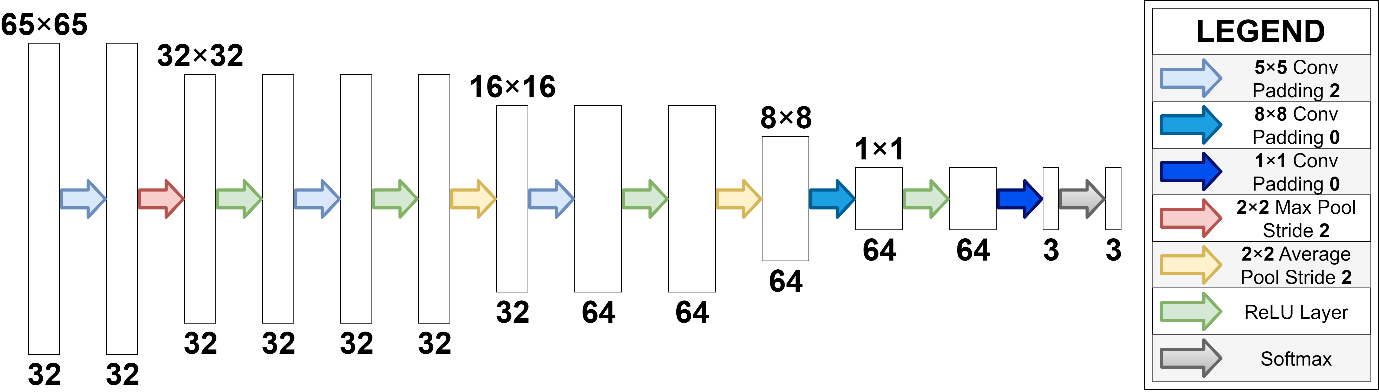


**Supplementary Figure 1** The CNN network architecture used in this study.
